# Risk factors for endocrinological immune-related adverse events in patients with renal cell carcinoma treated with immune checkpoint inhibitors

**DOI:** 10.1007/s40618-025-02732-z

**Published:** 2025-11-10

**Authors:** Nicola Marrano, Mariangela Caporusso, Carlo Ganini, Andrea Borraccino, Angelo Cignarelli, Sebastio Perrini, Luigi Laviola, Camillo Porta, Francesco Giorgino, Annalisa Natalicchio

**Affiliations:** 1 Department of Precision and Regenerative Medicine and Ionian Area, Section of Internal Medicine, Endocrinology, Andrology and Metabolic Diseases, University of Bari Aldo Moro, Bari, Italy; 2 Endocrinology Unit, Regional General Hospital “Francesco Miulli”, Acquaviva Delle Fonti, Bari, Italy; 3 Interdisciplinary Department of Medicine, Division of Medical Oncology, University of Bari Aldo Moro, Bari, Italy; 4 Department of Medicine and Surgery, Section of Endocrinology, LUM University, Casamassima, Bari, Italy; 5 Section of Internal Medicine, Endocrinology, Andrology and Metabolic Diseases, Department of Precision and Regenerative Medicine and Ionian Area, University of Bari Aldo Moro, Piazza Giulio Cesare, 11, 70124 Bari, Italy

**Keywords:** Renal cell carcinoma, Endocrine immune-related adverse events, Immune checkpoint inhibitors, Anti-angiogenic agent

## Abstract

**Purpose:**

First-line treatment for renal cell carcinoma (RCC) commonly includes immune checkpoint inhibitors (ICIs), either as monotherapy or in combination with anti-angiogenic agents. These therapies can lead to endocrine immune-related adverse events (irAEs). This study aimed to identify predictive risk factors for the development of endocrine irAEs associated with ICI-based therapies.

**Methods:**

We conducted an observational, retrospective, single-center study involving 72 patients with RCC who initiated first-line treatment with ICIs (either in combination with another ICI or an anti-angiogenic agent) between January 2018 and May 2023. All patients had at least 18 months of clinical and biochemical follow-up.

**Results:**

51.39% of patients experienced endocrine irAEs, including thyroid dysfunction (89.2%), primary hypocortisolism (10.8%), and hypophysitis (8.1%). Patients who developed endocrine irAEs had a significantly lower International Metastatic RCC Database Consortium (IMDC) score (p = 0.033), and renal/adrenal metastases were significantly associated with a lower risk of developing endocrine irAEs (p = 0.045) and thyroid dysfunction (p = 0.026). TNM stage II and III at diagnosis were linked with higher rates of endocrine irAEs in males and thyroid dysfunction in the overall population, whereas TNM stage IV was associated with a lower incidence of both outcomes (p = 0.02 and p = 0.05, respectively). In logistic regression analysis of the interaction between stage and sex, TNM stage III was significantly associated with a higher risk of irAEs and thyroid dysfunction in men compared with women at the same stage (p = 0.0184 and p = 0.0301, respectively). Among treatment variables, the use of tyrosine kinase inhibitors (TKIs) emerged as a significant predictor of thyroid irAEs (p = 0.041). A neutrophil percentage below the cohorts’ 50th percentile (61.45%) was associated with increased risk of endocrine irAEs (p = 0.048). In multivariate analysis, renal/adrenal metastases and TNM stage IV remained negative predictors of both endocrine irAEs and thyroid dysfunction, while TKI use was a significant positive predictor of thyroid dysfunction.

**Conclusions:**

This study highlights several significant associations between the occurrence of endocrine irAEs and oncological parameters (renal/adrenal metastases, TNM stage), therapeutic factors (use of TKIs), and laboratory markers (neutrophil percentage) in RCC patients. These predictors may be useful in identifying patients who are more likely to develop endocrine irAEs and therefore require more rigorous endocrine surveillance during treatment.

**Supplementary Information:**

The online version contains supplementary material available at10.1007/s40618-025-02732-z.

## Introduction

Renal cancer is the 14th most common malignancy worldwide, with over 430.000 new cases diagnosed in 2020 [1]. Renal cell carcinoma (RCC) accounts for approximately 90% of all renal cancers [1–3], and most cases are diagnosed accidentally. Notably, around one-third of RCC cases are diagnosed at an advanced or metastatic stage (mRCC) [4]. Over the past three decades, the treatment landscape of mRCC has undergone substantial changes with the introduction of vascular endothelial growth factor (VEGF)-targeting agents (anti-angiogenic therapies) and immune checkpoint inhibitors (ICIs). These therapies have significantly improved both progression-free survival (PFS) and overall survival (OS), establishing a new standard of care [5]. Consequently, while RCC incidence rates have been rising, mortality rates have shown a gradual decline [1, 2]. In particular, for the treatment of mRCC, the dual ICI combination targeting cytotoxic T lymphocyte-associated antigen 4 (CTLA-4) and programmed cell death 1 (PD-1) protein (ICI + ICI combination, e.g., ipilimumab + nivolumab) and the combination of an anti-PD1 ICI plus a vascular endothelial growth factor (VEGF) tyrosine kinase inhibitor (ICI + TKI combination, e.g., nivolumab + cabozantinib, pembrolizumab + axitinib, pembrolizumab + lenvatinib) have been approved as first-line therapy [2, 5].

Although the efficacy of ICIs in the treatment of mRCC is well established, their use has been associated with a distinct spectrum of immune-related adverse events (irAEs), affecting almost every organ of the body including the endocrine system [6, 7]. Endocrine toxicities are relatively common and may involve thyroid, parathyroids, pituitary, adrenal, and pancreas, resulting in hypothyroidism, hyperthyroidism, thyroid eye disease, hypoparathyroidism, hypophysitis, adrenal insufficiency, and diabetes mellitus [8]. While endocrine irAEs typically emerge within 3 to 6 weeks of initiating therapy, they may occur at any point during treatment, even after its cessation [9, 10]. Notably, recent studies indicate that endocrine toxicities are irreversible in approximately 50% of cases [7] and can be life-threatening if not promptly recognized and appropriately managed [6, 11]. Delays in diagnosis are common and may necessitate the discontinuation of ICI therapy [6].

The identification of reliable and validated biomarkers capable of predicting the occurrence of endocrine irAEs is thus highly desirable. Such markers could guide more rigorous and frequent endocrinological monitoring in selected patients, enabling earlier diagnosis and intervention. This, in turn, would improve patients’ quality of life and support the continuation of potentially life-prolonging anti-tumor therapies. To date, no biomarkers have been definitively validated as predictors of irAEs in patients treated with ICIs [12], even though some clinical and biochemical factors have been associated with an increased risk of endocrine irAEs [13]. For example, the presence of pre-existing antithyroid antibodies [14–16] and elevated baseline TSH levels [17–20] have been linked to a higher likelihood of ICI-induced thyroiditis. Additionally, alterations in baseline blood cell counts, including neutrophils, lymphocytes, monocytes, eosinophils, basophils, and platelets, as well as increases in lymphocyte and eosinophil counts during treatment, and various blood cell ratios (e.g., neutrophil-to-lymphocyte ratio), have been associated with increased irAE risk [13]. Other potential biomarkers (such as proinflammatory cytokines, antinuclear antibodies, anti-double-stranded DNA antibodies, microRNAs, genetic variants, human leukocyte antigen genotyping, and characteristics of the gastrointestinal microbiome) have been proposed, though many remain under investigation or are not yet routinely available in clinical practice [13].

In this retrospective study, we aimed to identify potential risk factors that may predict the development of endocrine irAEs in patients with mRCC undergoing treatment with ICIs.

## Methods

### Study design and population

This was a monocentric, observational, retrospective cohort study conducted at the Division of Medical Oncology, University Hospital Policlinico Consorziale of Bari. The study included adult patients diagnosed with RCC who initiated first-line treatment with a combination of ICIs or an ICI in combination with an anti-angiogenic agent between January 2018 and May 2023. The study protocol was approved by the Ethics Committee of the University Hospital Policlinico Consorziale of Bari (Document number: 7823, date: 15 May 2024).

Eligible patients met the following inclusion criteria: age ≥ 18 years, diagnosis of RCC, first-line treatment with either dual ICI therapy or a combination of an ICI and an anti-angiogenic agent. Both patients who developed and those who did not develop endocrine irAEs were included. Endocrine irAEs considered in this study included thyroid dysfunction, hypoparathyroidism, hypophysitis, primary adrenal insufficiency, and immune-related diabetes mellitus. Exclusion criteria included a history of pre-existing endocrine disorders prior to the initiation of ICI therapy, and prior exposure to other oncologic treatments before the first ICI administration. All information was retrospectively retrieved from available records.

Patients were considered enrolled on the date of their first ICI treatment cycle (baseline), between January 1, 2018, and May 31, 2023. They were subsequently categorized based on whether they developed endocrine irAEs within 18 months of treatment initiation, with the latest observation point being November 30, 2024.

### Data collection

The clinical data of patients were collected by consulting the electronic medical record system. At baseline (pre-treatment), demographic (age, sex, ethnicity), anthropometric (weight, height, BMI), laboratory (complete blood count, creatinine, calcium, glycemia, albumin, TSH, and all available blood parameters), and clinical (tumor type, disease duration, oncologic therapy details, disease course) data were collected. Additional data included smoking status, alcohol consumption, family history of endocrine disorders, prior endocrinopathies, and previous malignancies. For patients who developed endocrine irAEs, specific information on the type of endocrinopathy and the time to onset from ICI initiation was recorded. ICI-induced endocrine irAEs were defined as follows: primary thyroid dysfunction, including both hypothyroidism (diagnosed on high TSH levels with low/normal fT3, fT4, requiring medical intervention) and hyperthyroidism (defined as low TSH levels with high/normal fT3, fT4, also requiring medical intervention); primary adrenal insufficiency, diagnosed by the presence of very low morning cortisol levels, with elevated ACTH levels and suggestive symptoms, confirmed by inadequate response to the ACTH stimulation test in patients with indeterminate serum morning cortisol levels; hypophysitis, defined by low pituitary hormone levels and typical magnetic resonance imaging (MRI).

For patients with complete data availability, the International Metastatic RCC Database Consortium (IMDC) score was calculated. This prognostic score predicts the survival outcomes of patients with metastatic RCC undergoing systemic therapy by incorporating six adverse prognostic factors present at baseline (pre-treatment): time from diagnosis to initiation of systemic therapy < 1 year, Karnofsky Performance Status < 80%, haemoglobin below the lower limit of normal, corrected serum calcium level above the upper limit of normal, absolute neutrophil count above the upper limit of normal, and platelet count above the upper limit of normal [21]. Each factor is assigned a score of 1 if present and 0 if absent. The sum of these values categorizes patients into three prognostic groups: a total score of 0 defines the “good risk” group, 1–2 defines the “intermediate risk” group, and 3–6 the “poor risk” group of patients. This scoring system was used in the present study to explore associations between baseline risk profile and the development of endocrine irAEs.

For patients who experienced one or more endocrine irAEs, severity was graded according to the Common Terminology Criteria for Adverse Events (CTCAE, version 5.0) and classified on a scale from Grade 1 (G1) to Grade 5 (G5).

### Statistical analysis

Continuous variables were summarized as means with standard deviations or medians with interquartile ranges (IQRs), depending on data distribution. Categorical variables were reported as frequencies and percentages. Patients were categorized into two groups based on the occurrence of endocrine irAEs: those who developed endocrine irAEs and those who did not. Only for variables that showed statistically significant differences in the primary analysis, a subgroup analysis was additionally performed for thyroid dysfunction. Exploratory subgroup analyses were conducted by sex. Group comparisons for continuous variables were conducted using the independent samples t-test for normally distributed data, and the Mann–Whitney U test for non-normally distributed data. Categorical variables were compared using the chi-square test. PFS and OS based on endocrine irAE occurrence, as well as the time to onset of endocrine irAEs or thyroid dysfunction according to the significant association identified, were analysed using the log-rank test and Kaplan–Meier survival curves were generated. For significant log-rank results, Cox-proportional hazard test was conducted to estimate Hazard Ratios (HR). Both chi-square and Cox-proportional hazard tests were performed, as the chi-square test assesses differences in categorical distributions between groups, whereas the Cox-proportional hazard test is specifically designed for time-to-event data, accounting for both the occurrence and timing of events, thus providing a more accurate assessment in survival analyses.

## Results

**Fig. 1:**
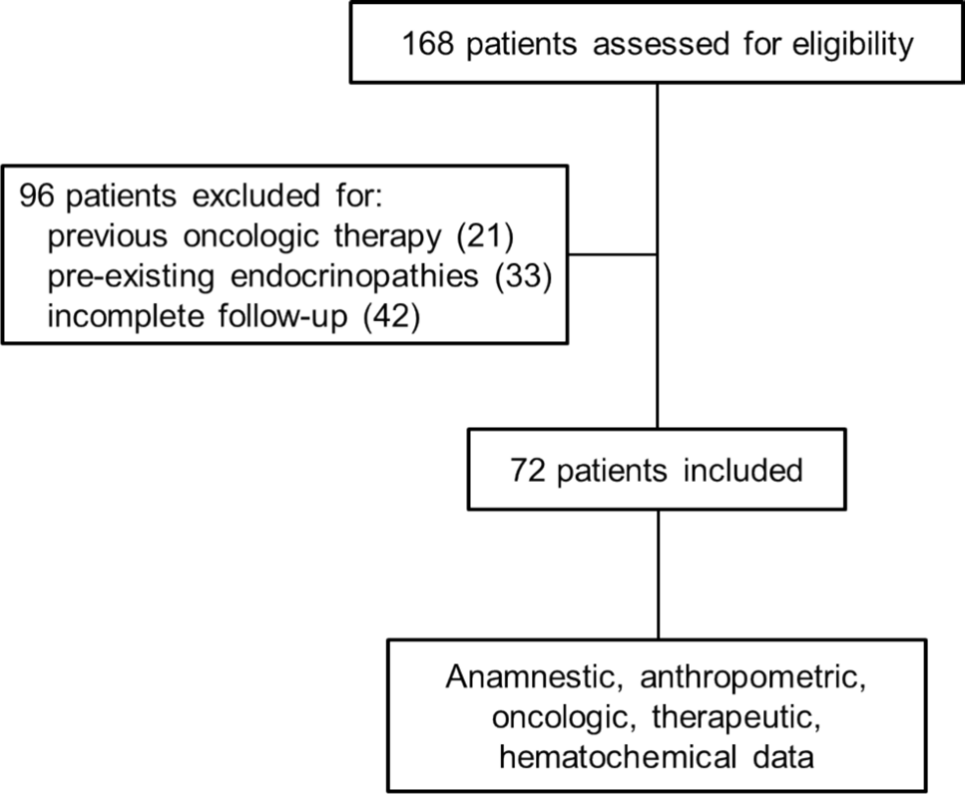
Flow diagram of the study

A total of 168 patients were initially screened for eligibility. After excluding 96 patients who did not meet the inclusion criteria (due to previous oncologic therapy, pre-existing endocrinopathies, or incomplete follow-up), 72 patients were enrolled in the study (Fig. 1). The mean age of the cohort was 57.44 years, and the majority were male (70.83%) (Table 1). Most patients (81.94%) received a combination of ICI and anti-angiogenic therapy. Among these, 80.56% were treated with TKIs (62.07% with axitinib, 17.24% with lenvatinib, 13.79% with cabozantinib, 3.45% with axitinib followed by lenvatinib, 3.45% axitinib followed by cabozantinib), with or without belzutifan, a hypoxia-inducible factor-2α (HIF-2α) inhibitor, and 9.44% received belzutifan monotherapy. The remaining 18.06% of patients were treated with dual ICI therapy (Table 2). At the time of diagnosis, most patients (41.94%) had TNM stage IV tumors, followed by 35.48% with stage III, 19.36% with stage II, and 3.23% with stage I (Table 2). Nearly all patients (97.22%) presented with at least one metastatic site. The most frequent metastatic location included lung (65.28%), lymph nodes (37.5%), bone (27.78%), liver (20.83%), soft tissues (18.06%), renal/adrenal gland (16.67%), brain (6.94%), intestine (4.17%), and pancreas (2.78%) (Table 2). The predominant histological subtype was clear cell renal carcinoma (81.16%), followed by papillary (8.7%), chromophobe (4.35%), and other subtypes (5.8%) (Table 2).

**Table 1:** Anamnestic and anthropometric parameters of the enrolled population

Parameter	N	Total population (n = 72)	No (e)irAEs (n = 35)	(e)irAEs (n = 37)	p-value
Age (years)	72	57.44 (12.01)	58.29 (11.65)	56.65 (12.46)	0.567
Weight (kg)	55	75.05 (15.67)	73.23 (14.07)	76.26 (16.75)	0.487
BMI (Kg/m^2^)	35	25.33 (3.96)	24.85 (3.82)	25.687 (4.12)	0.546
Male	72	51 (70.83)	25 (49.02)	26 (50.98)	0.914
Alcohol users	72	42 (58.33)	21 (50)	21 (50)	0.780
Smokers	72	37 (51.39)	17 (45.95)	20 (54.05)	0.642
History of diabetes	72	10 (13.89)	7 (70)	3 (30)	0.145
History of thyroid nodules	72	5 (6.94)	3 (60)	2 (40)	0.597
History of autoimmune diseases	63	6 (8.33)	2 (33.33)	4 (66.67)	0.286

Continuous variables are expressed as mean (standard deviation); categorical variables are expressed as n (%). BMI: body mass index; irAEs: immune-related adverse events

**Table 2:** Oncologic and therapeutic parameters of the enrolled population divided according to endocrine irAEs occurrence

Parameter	N	Total population (n = 72)	No (e)irAEs (n = 35)	(e)irAEs (n = 37)	p-value
**Histology**	69				0.561
Clear cell		56 (81.16)	29 (51.79)	27 (48.21)	
Papillary		6 (8.7)	2 (33.33)	4 (66.67)	
Chromophobe		3 (4.35)	1 (33.33)	2 (66.67)	
Other		4 (5.8)	3 (75)	1 (25)	
**TNM stage**	62				0.105
I		2 (3.23)	1 (50)	1 (50)	
II		12 (19.36)	3 (25)	9 (75)	
III		22 (35.48)	9 (40.91)	13 (59.09)	
IV		26 (41.94)	17 (65.39)	9 (34.61)	
**TNM stage**	62				
***Males***	44				**0.02**
I		2 (4.56)	1 (50)	1 (50)	
II		8 (18.18)	2 (25)	6 (75)	
III		16 (36.36)	5 (31.25)	11 (68.75)	
IV		18 (40.91)	14 (77.78)	4 (22.22)	
***Females***	18				0.374
I		0	0	0	
II		4 (22.22)	1 (25)	3 (75)	
III		6 (33.33)	4 (66.67)	2 (33.33)	
IV		8 (44.44)	3 (37.5)	5 (62.5)	
**Nephrectomy**	71	51 (71.83)	23 (45.1)	28 (54.9)	0.259
**Site of metastases**	72				
Lung		47 (65.28)	23 (48.94)	24 (51.06)	0.94
Lymph node		27 (37.5)	15 (55.56)	12 (44.44)	0.361
Bone		20 (27.78)	8 (40)	12 (60)	0.365
Liver		15 (20.83)	6 (40)	9 (60)	0.453
Soft tissue		12 (18.06)	5 (38.46)	8 (61.54)	0.419
Renal/adrenal		12 (16.67)	9 (75)	3 (25)	**0.045**
Brain		5 (6.94)	3 (60)	2 (40)	0.597
Intestine		3 (4.17)	1 (33.33)	2 (66.67)	0.589
Pancreas		2 (2.78)	0	2 (100)	0.163
**IMDC score**	41 (21;20)	2 (1–2)	2 (1–3)	1 (0.75–2)	0.033*
**ICI + ICI**	72	13 (18.06)	8 (61.54)	5 (38.46)	0.303
**ICI + anti-angiogenic**	72	59 (81.94)	27 (45.76)	32 (54.24)	0.303
**TKIs**	72	58 (80.56)	26 (44.82)	32 (55.17)	0.191

Continuous variables are expressed as median (interquartile range), categorical variables are expressed as n (%). Statistically significant p-values are highlighted in bold. *Mann–Whitney test; (e)irAEs: endocrine immune-related adverse events; ICI: immune check-point inhibitor; IMDC: International Metastatic RCC Database Consortium; TKIs: tyrosine kinase inhibitors

Of the 72 patients, 51.39% developed at least one endocrine irAE, and 19% experienced two or more endocrine events (Table 3). The most frequently observed endocrine irAE was thyroid dysfunction, affecting 89.2% of those with endocrine irAEs. Among these, 86.5% developed primary hypothyroidism, while 13.5% developed hyperthyroidism (Table 3). Additionally, 10.8% of patients developed primary hypocortisolism and 8.1% experienced hypophysitis (Table 3). The median follow-up was 57.75 weeks (IQR 31.78–110.6) and the median time to onset of endocrine irAEs was 13.86 weeks for hypophysitis, 18.72 weeks for thyroid dysfunction, and 39.22 weeks for primary hypocortisolism. According to CTCAE v5.0, 37.8% of endocrine irAEs were classified as G1, 48.7% as G2, and 5.4% as G3 (Table 3). Moreover, 5.4% of patients experienced both thyroid irAEs and primary hypocortisolism, with G1 toxicity for both events, while 2.7% had G2 thyroid irAEs accompanied by G1 primary hypocortisolism (Table 3).

**Table 3:** Endocrine irAEs description

Parameter	N	N (%)	Median time to onset (weeks)
**(e)irAEs**	72	37 (51.39)	18.29 (11.86–35.782)
≥ 2 events	72	7 (19)	
**Thyroid dysfunction**	72	33 (89.2)	18.72 (11.04–35.78)
Hypothyroidism		32 (86.5)	19 (11.72–35.86)
Hyperthyroidism		5 (13.5)	13.645 (6.86–19.72)
**Primary hypocortisolism**	72	4 (10.8)	39.22 (27.36–50.68)
**Hypophysitis** MRI confirmation	72	3 (8.1) 3 (8.1)	13.86 (13.15.36)
**CTCAE** G1 G1 thyr, G1 hypoc G2 G2 thyr, G1 hypoc G3	37	14 (37.8) 2 (5.4) 18 (48.7) 1 (2.7) 2 (5.4)	13.79 (9.54–13.79) 39.22 (34.54–39.22) 18.14 (13.86–18.14) 19.86 28 (21–28)

(e)irAEs: endocrine immune-related adverse events, CTCAE: Common Terminology Criteria for Adverse Events; CTCAE hypoc: primary hypocortisolism; CTCAE thyr: refers to thyroid irAEs; MRI: magnetic resonance imaging

### Association between anamnestic and anthropometric parameters and endocrine irAEs occurrence

No statistically significant differences were observed between patients who developed endocrine irAEs and those who did not with respect to anamnestic or anthropometric parameters, such as age at diagnosis, weight, BMI, sex, smoke and alcohol habits, history of diabetes, thyroid nodules and autoimmune diseases (Table 1). We only observed a trend toward higher CTCAE grades in younger patients (61.07 ± 10.4 years G1 vs 53.06 ± 14.31 G2, p = 0.088).

### Association between oncologic parameters and endocrine irAEs occurrence

Among oncologic characteristics, the presence of renal/adrenal metastases was significantly associated with a lower incidence of endocrine irAEs. Specifically, only 25% of patients with renal/adrenal metastases developed endocrine irAEs, compared to 56.7% of those without such metastases (p = 0.045; Table 2). A similar result was observed for thyroid dysfunction, which was significantly less frequent in patients with renal/adrenal metastases (p = 0.026; Table 4). However, these associations were not confirmed by log-rank analysis (p = 0.19 for endocrine irAEs and p = 0.12 for thyroid dysfunction).

**Table 4:** Oncologic and therapeutic parameters of the enrolled population according to thyroid dysfunction occurrence

Parameter	N	Total population (n = 72)	Thyroid dysfunction (n = 39)	No thyroid dysfunction (n = 33)	p-value
**Renal/adrenal metastases**	72	12 (16.67)	10 (83.33)	2 (16.67)	**0.026**
**TNM stage**	62				**0.05**
I		2 (3.23)	2 (100)	0	
II		12 (19.36)	3 (25)	9 (75)	
III		22 (35.48)	10 (45.56)	12 (54.55)	
IV		26 (41.94)	17 (65.39)	9 (34.62)	
**TNM stage**	62				
***Males***	44				**0.016**
I		2 (4.56)	2 (100)	0	
II		8 (18.18)	2 (25)	6 (75)	
III		16 (36.36)	6 (37.5)	10 (62.5)	
IV		18 (40.91)	14 (77.78)	4 (22.22)	
***Females***	18				0.374
I		0	0	0	
II		4 (22.22)	1 (25)	3 (75)	
III		6 (33.33)	4 (66.67)	2 (33.33)	
IV		8 (44.44)	3 (65.7)	5 (32.5)	
**IMDC score**	41 (21;20)	2 (1–2)	2 (1–3)	1 (1–2)	0.094*
**TKIs** Axitinib/cabozantinib Axitinib/lenvatinib Axitinib Lenvatinib Cabozantinib	72	58 (80.56) 2 (3.45) 2 (3.45) 36 (62.07) 10 (17.24) 8 (13.79)	28 (48.28) 16 (44.44) 5 (50) 4 (50)	30 (51.72) 20 (55.56) 5 (50) 4 (50)	**0.041** 0.928

Continuous variables are expressed as median (interquartile range), categorical variables are expressed as n (%). Statistically significant p-values are highlighted in bold. *Mann–Whitney test; ICI: immune check-point inhibitor; IMDC: International Metastatic RCC Database Consortium; TKIs: tyrosine kinase inhibitors

Although the overall distribution of endocrine irAEs did not differ significantly by TNM stage, an exploratory sex-specific subgroup analysis revealed significant associations in male patients. In this subgroup, endocrine irAEs occurred more frequently in those with stage II and III tumors and were less common in those with stage IV tumors (p = 0.02; Table 2). Specifically, 77.78% of male patients with TNM stage IV tumors did not develop endocrine irAEs, whereas 75% and 68.75% of those with stage II and stage III tumors, respectively, did (Table 2). This trend was not confirmed in female patients. Similarly, thyroid dysfunction was more prevalent in patients with stage II tumors and less frequent in those with stage IV tumors (p = 0.05) in the overall population. This trend was confirmed in male patients (p = 0.016; Table 4), but not in female patients. To account for multiple comparisons, we performed a logistic regression analysis including the interaction between sex and disease stage. We found the risk of irAEs and thyroid dysfunction occurrence was significantly higher in men with stage III disease vs women with the same stage (OR = 25.67, 95% CI: 1.87–449.21, p = 0.0184; OR = 19.4, 95% CI: 1.44–333.749, p = 0.0301, Supplementary Tables S1 and S2). Moreover, the association between TNM stage and both endocrine irAEs and thyroid dysfunction did not reach statistical significance in the log-rank analysis (p = 0.36 and p = 0.33, respectively).

**Fig. 2:**
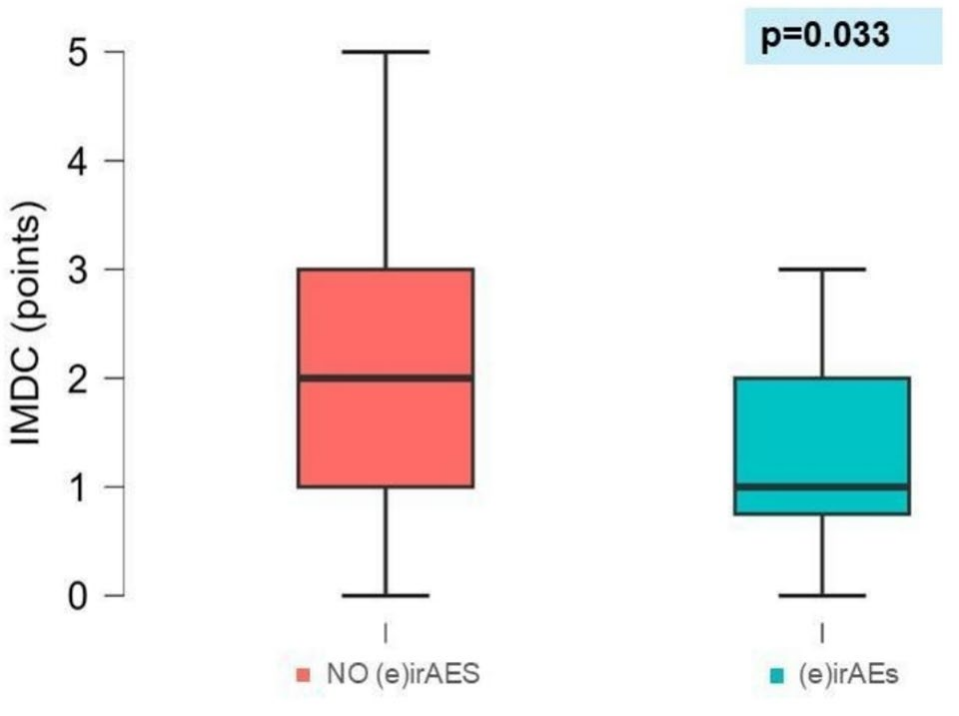
Association between IMDC score and endocrine irAEs. Box plot of the International Metastatic RCC Database Consortium (IMDC) score in patients with and without endocrine immune-related adverse events [(e)irAEs)]. Patients who developed endocrine irAEs (cyan) had a significantly lower IMDC score compared to those without endocrine irAEs (red) (p = 0.033), suggesting a potential association between irAEs and better prognostic features. Values are reported as median (Interquartile Range), Mann–Whitney p-value

Patients who developed endocrine irAEs also had significantly lower International Metastatic RCC Database Consortium (IMDC) scores compared to those who did not [median IMDC score: 1 (IQR 0.75–2) vs. 2 (IQR 1–3); p = 0.033] (Table 2, Fig. 2). In contrast, the IMDC score was not significantly associated with thyroid dysfunction (p = 0.094; Table 4).

**Fig. 3:**
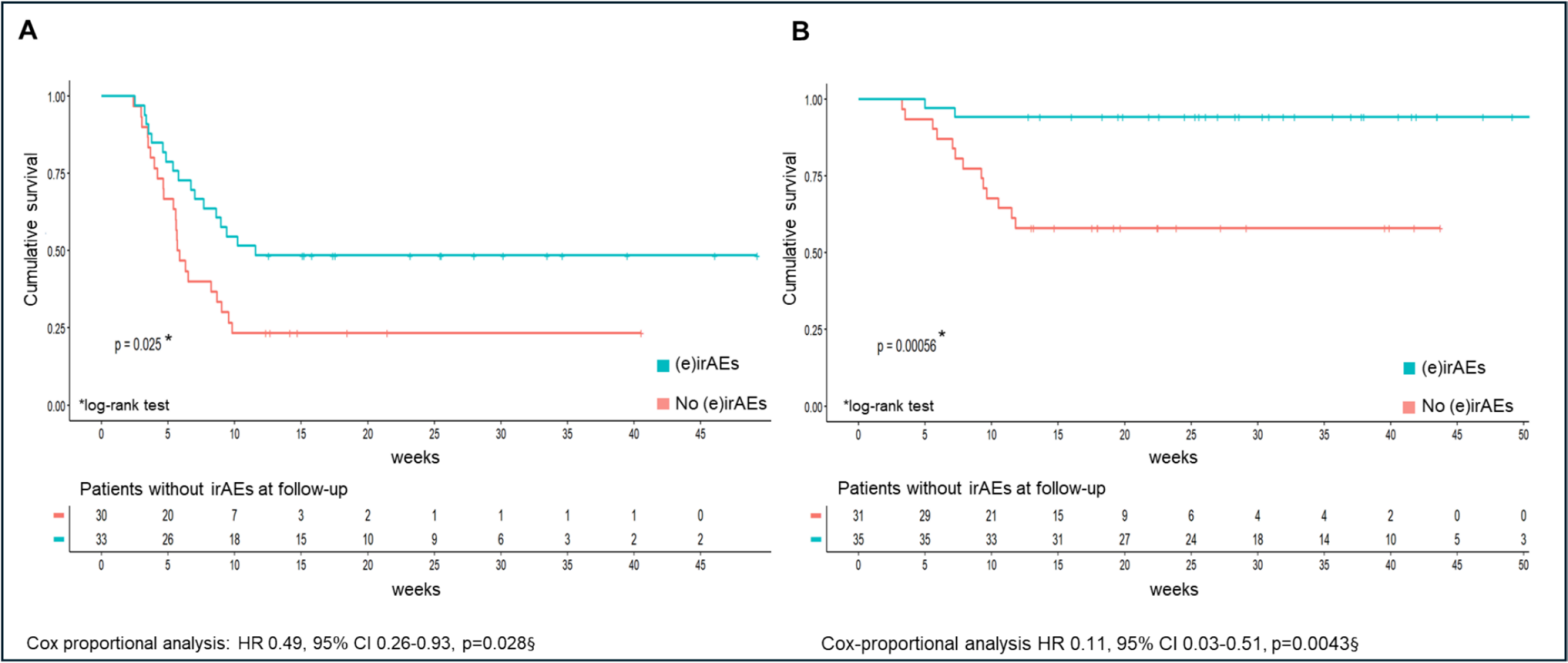
Progression Free Survival and Overall Survival according to endocrine irAEs**.** Kaplan–Meier survival curves for progression-free survival (PFS, panel **A**) and overall survival (OS, panel **B**) in patients with or without endocrine immune-related adverse events [(e)irAEs]. Patients experiencing endocrine irAEs had improved survival outcomes compared to those without endocrine irAEs. Panel A: § unadjusted; adjusted for disease stage at the time of kidney cancer diagnosis (I + II vs. III + IV): HR = 0.33, 95%CI: 0.15–0.71, p = 0.0047; adjusted for disease stage at the time of kidney cancer diagnosis + treatment modality (ICI + ICI vs. ICI + TKI): HR = 0.32, 95% CI: 0.15–0.73, p = 0.006; adjusted for disease stage at the time of kidney cancer diagnosis + treatment modality + IMDC score (favourable vs. intermediate vs. poor): HR = 0.45, 95% CI: 0.11–1.71, p = 0.24. Panel B: §unadjusted; adjusted for disease stage at the time of kidney cancer diagnosis (I + II vs. III + IV): HR = 0.12, 95% CI: 0.03–0.59, p = 0.009; adjusted for disease stage at the time of kidney cancer diagnosis + treatment modality (ICI + ICI vs. ICI + TKI): HR = 0.13, 95%CI: 0.003–0.63, p = 0.012; adjusted for disease stage at the time of kidney cancer diagnosis + treatment modality + IMDC score (favourable vs. intermediate vs. poor): HR = 0.08, 95% CI: 0.002–2.3, p = 0.138

Finally, survival analysis showed that both 12-month progression-free survival (PFS; Fig. 3A) and overall survival (OS; Fig. 3B) were significantly improved in patients who developed endocrine irAEs, assessed by the log-rank test (PFS: p = 0.025; OS: p = 0.0056) and univariate Cox proportional analysis (PFS: HR = 0.49, 95% CI: 0.26–0.93, p = 0.028; OS: HR = 0.11, 95% CI 0.03–0.51, p = 0.0043). After adjustment for disease stage at the time of kidney cancer diagnosis (I + II vs III + IV), and treatment type (ICI + ICI vs ICI + TKI) both PFS and OS remained significantly improved in patients who developed irAEs. However, when further adjusting for stage at the time of cancer diagnosis, treatment, and IMDC risk category (favorable vs intermediate vs poor), no significant differences in PFS or OS were observed between patients with and without irAEs. This result should be interpreted with caution, as IMDC data was available only for a minority of patients (Tables 5 and 6).

**Table 5:** Univariate and multivariate Cox-regression analysis for 12-month PFS according to the presence of irAEs

Variables	Hazard Ratio (95% CI)	p-value	likelihood p-value	n. of observations	n. of events
Model 1 (Unadjusted)	0.49 (0.26–0.93)	0.028	0.03	63	40
Model 2*	0.33 (0.15–0.71)	0.0047	0.006	53	31
Model 3§	0.32 (0.15–0.73)	0.006	0.02	52	31
Model 4#	0.45 (0.11–1.71)	0.24	0.0004	29	7

^*^Adjusted for disease stage at the time of kidney cancer diagnosis (I + II vs. III + IV); § adjusted for disease stage at the time of kidney cancer diagnosis + treatment modality (ICI + ICI vs. ICI + TKI); # adjusted for disease stage at the time of kidney cancer diagnosis + treatment modality + IMDC score (favourable vs. intermediate vs. poor). PFS: progression free survival; IMDC: International Metastatic RCC Database Consortium; ICI: immune check-point inhibitor; TKI: tyrosine kinase inhibitor; irAEs: immune-related adverse events; CI: confidence interval

**Table 6:** Univariate and multivariate Cox-regression analysis for OS according to the Presence of irAEs

Variables	Hazard ratio (95% CI)	p-value	likelihood p-value	n. of observations	n. of events
Model 1 (Unadjusted)	0.11 (0.03–0.51)	0.0043		66	15
Model 2*	0.12 (0.03–0.59)	0.009	0.006	56	13
Model 3§	0.13 (0.003–0.63)	0.012	0.01	55	13
Model 4#	0.08 (0.002–2.3)	0.138	0.006	31	8

^*^Adjusted for disease stage at the time of kidney cancer diagnosis (I + II vs. III + IV); § adjusted for disease stage at the time of kidney cancer diagnosis + treatment modality (ICI + ICI vs. ICI + TKI); # adjusted for disease stage at the time of kidney cancer diagnosis + treatment modality + IMDC score (favourable vs. intermediate vs. poor). OS: overall survival; IMDC: International Metastatic RCC Database Consortium; ICI: immune check-point inhibitor; TKI: tyrosine kinase inhibitor; irAEs: immune-related adverse events; CI: confidence interval

### Association between therapeutic parameters and endocrine irAEs occurrence

**Fig. 4:**
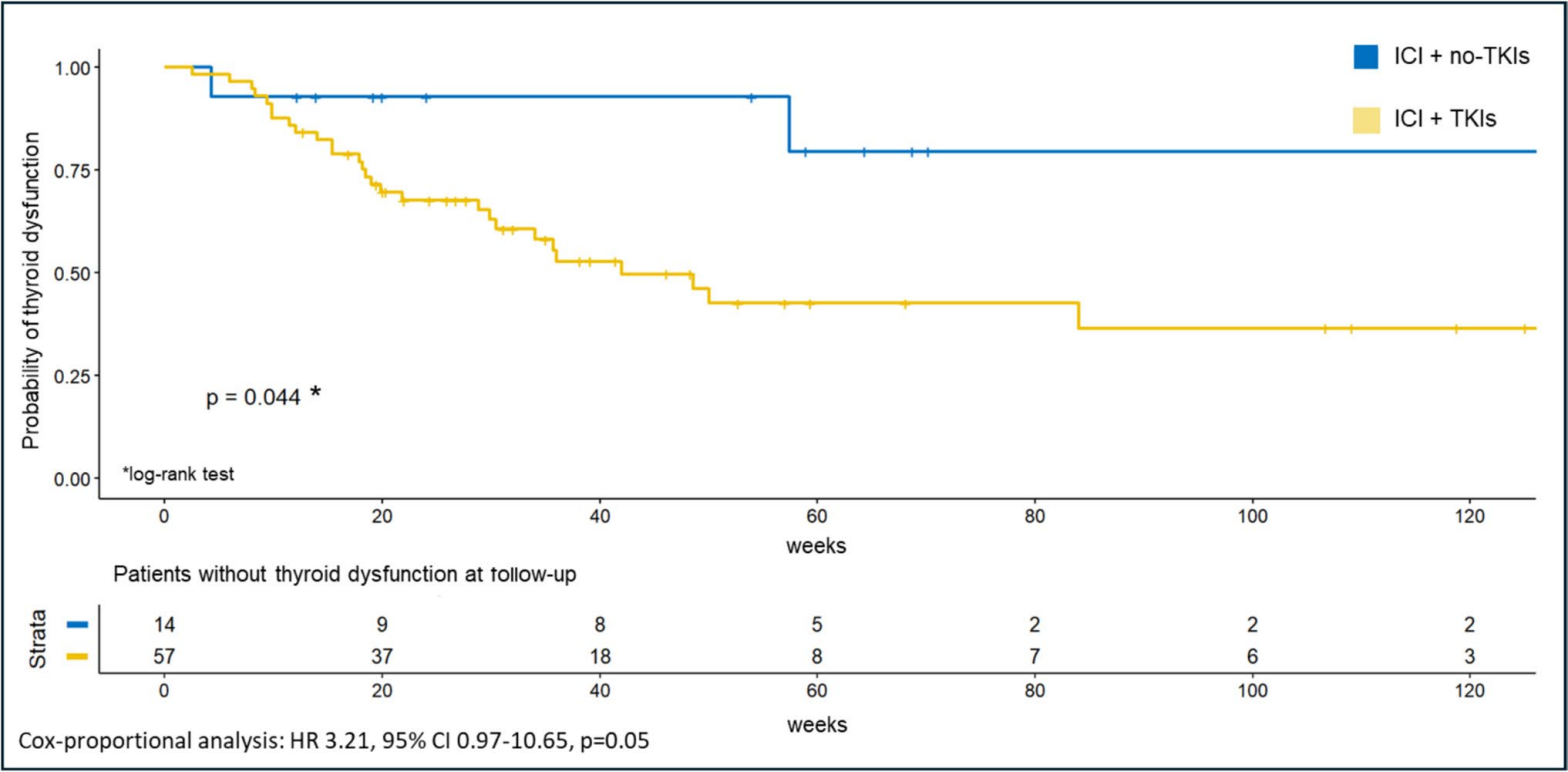
Probability of thyroid dysfunction according to therapy. Kaplan–Meier curve showing the probability of thyroid dysfunction in patients receiving immune checkpoint inhibitors (ICI) in combination with tyrosine kinase inhibitor (TKI) or no-TKI anti-angiogenic agent (i.e. belzutifan); patients receiving TKIs had a significantly higher probability of thyroid dysfunction occurrence (p = 0.044)

No significant difference in the overall incidence of endocrine irAEs was observed between patients treated with ICI + TKI combinations and those receiving ICI + a non-TKI anti-angiogenic agent (i.e., belzutifan). This was confirmed both in the chi-square test (p = 0.191) and log-rank survival analysis (p = 0.13) (Table 2). However, a significant association was found between the use of ICI + TKI combination and the development of thyroid dysfunction compared to patients receiving ICI + a non-TKI anti-angiogenic agent. Specifically, thyroid dysfunction occurred in 21.43% of patients not treated with TKIs, compared to 51.72% of those receiving TKI-based regimens (Table 4; p = 0.041), regardless of TKI agent used. Notably, 90.91% of patients who developed thyroid dysfunction had been treated with TKIs. Furthermore, the log-rank analysis confirmed that thyroid dysfunction occurred more frequently and with an earlier onset in patients receiving TKIs (log-rank test p = 0.044; Cox-proportional test: HR = 3.21, 95% CI: 0.97–10.65, p = 0.05; Fig. 4).

### Association between biochemical parameters and endocrine irAEs occurrence

Among biochemical parameters, we found that endocrine irAEs were significantly more prevalent among patients with pre-treatment neutrophil percentage < 50th centile of the cohort (61.45%, p = 0.048) (Table 7). This association was not confirmed at log-rank analysis (p = 0.17). Additionally, we analyzed the neutrophil-to-lymphocyte ratio (NLR) and found no significant association [patients with irAEs: NLR 2.854 (1.616–9.4); patients without irAEs: NLR 3.451 (1.218–8.385); Mann–Whitney p = 0.463].

**Table 7:** Biochemical parameters of the enrolled population

Parameter	N	Total population (n = 72)	No (e)irAEs (n = 35)	(e)irAEs (n = 37)	p-value
**RBC (million/m**^**3**^)* < 4.5 million/m^3^	43 (22;21)	4.54 (4.25–5.18)	5.03 (4.32–5.19) 8 (44.44)	4.52 (4.24–5.12) 10 (55.56)	0.437 0.455
**Hb (g/dL)**	43 (22;21)	12.55 (2.08)	12.5 (2.24)	12.6 (1.96)	0.878
Anemia			13 (56.52)	10 (43.48)	0.451
**Neutrophils (%)**	43 (22;21)	61.45 (9.17)	68.78 (10.29)	67.85 (8.05)	0.743
< 61.45%			7 (35)	13 (65)	**0.048**
**Basophils (%)**	43 (22;21)	0.49 (0.29)	0.475 (0.314)	0.514 (0.29)	0.668
< 0.5%			12 (50)	12 (50)	0.864
**Lymphocytes (%)**	43 (22;21)	22.36 (7.85)	21.91 (8.88)	22.83 (6.79)	0.704
< 23.%			13 (54.17)	11 (45.83)	0.658
**Monocytes (%)**	43 (22;21)	6.47 (2.23)	6.36 (2.37)	6.58 (2.13)	0.754
< 6.5%			13 (59.09)	9 (40.91)	0.287
PLT (μL)*	43 (22;21)	266 (200.5–343)	330.5 (199–409.5)	263 (202–324)	0.313
< 266 ml			10 (45.46)	12 (54.54)	0.443
**NLR**	43 (22;21)	2.97 (2.34–4.37)	3.45 (1.22- 8.39)	2.85 (1.62–9.4)	0.463
**Creatinine (mg/dL)**	44 (22;22)	1.09 (0.32)	1.06 (0.331)	1.12 (0.313)	0.487
< 1.09 mg/dL			10 (45.45)	12 (54.54)	0.546
**Calcium (mg/dL)**	41 (21;20)	9.52 (0.69)	9.61 (0.78)	9.42 (0.59)	0.386
< 9.5 mg/dL			12 (52.17)	11 (47.83)	0.89
Glycemia (mg/dL)*	43 (22;21)	94 (84.5–114.5)	94 (80.75–124)	94 (86–101)	0.706
< 94 mg/dL			10 (47.62)	11 (53.38)	0.65
**Albumin (mg/dL)**	37 (18;19)	3.69 (0.87)	3.55 (0.9)	3.82 (0.85)	0.359
< 3.7 mg/dL			14 (53.85)	12 (46.15)	0.331
TSH (μU/mL)*	33 (13;20)	1.58 (1.15–3.18)	1.44 (1.08–3.29)	1.81 (1.26–3.06)	0.897
< 1.58 μU/mL			8 (47.06)	9 (52.94)	0.486

Continuous variables are expressed as mean (SD) or median (interquartile range), categorical variables are expressed as n (%); *values are expressed as median (interquartile range), Mann-Withney p-value. Statistically significant p-values are highlighted in bold. (e)irAEs: endocrine immune-related adverse events; Hb: haemoglobin; NLR: neutrophils-to-lymphocytes ratio; PLT: platelet; RBC: red blood cells

### Multivariate analysis

In the multivariate logistic regression analysis using a stepwise model, and after adjusting for confounding variables, the presence of renal/adrenal metastases (OR = 0.017, 95% CI: −7.364 to −0.733, p = 0.017) and TNM stage IV tumors (OR = 0.058, 95% CI: –5.364 to –0.338, p = 0.026) emerged as significant independent negative predictors of endocrine irAEs development (Table 8).

**Table 8:** Multivariate logistic regression analysis for predictors of endocrine irAEs

	Estimate	Standard error	Odds ratio	95% CI	p-value
(Intercept)	1.323	1.138	3.755	[–0.907; 3.553]	0.245
Neutrophils < 61.45%	1.648	0.919	5.199	[–0.152; 3.449]	0.073
Renal/adrenal metastases	–4.048	1.692	0.017	[–7.364; –0.733]	**0.017**
TNM stage III	–1.831	1.287	0.160	[–4.353; 0.691]	0.155
TNM stage IV	–2.851	1.282	0.058	[–5.364; –0.338]	**0.026**
Sex (female)	0.710	0.992	2.034	[–1.234; 2.654]	0.474

Statistically significant p-values are highlighted in bold. irAEs: immune-related adverse events; CI: confidence interval

Similarly, when analysing predictors of thyroid-specific irAEs, the presence of renal/adrenal metastases remained a significant negative predictor (OR = 0.063, 95% CI: −4.87 to −0.65, p = 0.011), while treatment with TKI was identified as a significant positive predictor (OR = 6.62, 95% CI: 0.18 to 3.6, p = 0.03) (Table 9).

**Table 9:** Multivariate logistic regression analysis for predictors of thyroid dysfunction

	Estimate	Standard error	Odds ratio	95% CI	p-value
(Intercept)	–15.652	1473.410	1.593 × 10⁻⁷	[–2903.483; 2872.179]	0.992
Sex (female)	0.484	0.688	1.622	[–0.865; 1.832]	0.482
TNM stage II	16.095	1473.411	9.769 × 10⁶	[–2871.737; 2903.927]	0.991
TNM stage III	14.611	1473.411	2.214 × 10⁶	[–2873.221; –2902.442]	0.992
TNM stage IV	13.480	1473.411	715,207.995	[–2874.352; –2901.312]	0.993
Renal/adrenal metastases	–2.757	1.078	0.063	[–4.869; –0.645]	**0.011**
TKIs	1.89	0.871	6.62	[0.183; 3.597]	**0.03**

Statistically significant p-values are highlighted in bold. irAEs: immune-related adverse events; CI: confidence interval

## Discussion

In the present study, we aimed to identify predictive risk factors for the development of ICI-related endocrine irAEs in a cohort of patients with RCC who initiated first-line treatment with ICIs, either in combination with another ICI (18.6%) or an anti-angiogenic agent (81.94%), at a single oncology centre. ICI-related endocrine irAEs occurred in 51.39% of patients, most commonly manifesting as thyroid dysfunction (89.2% of endocrine irAEs), with hypothyroidism being predominant (86.5%), followed by hyperthyroidism (13.5%). Less frequently, patients experienced primary adrenal insufficiency (10.8%) and hypophysitis (8.1%). These findings are consistent with previous studies, which have reported thyroid dysfunction as the most frequent endocrine toxicity associated with ICI therapy [22, 23].

In our cohort, the median time to onset was 18.72 weeks for thyroid dysfunction, 39.22 weeks for primary hypocortisolism, and 13.86 weeks for hypophysitis. These timeframes align with published data, as thyroid dysfunction typically arises within the first 15 weeks of treatment, though onset has been reported from as early as 7 days to as late as 3 years after initiation of therapy [8, 23, 24]; similarly, ICI-induced primary adrenal insufficiency has been reported to occur between 1 and 82 weeks [24, 25], while hypophysitis generally emerges between 8 and 10 weeks, with some cases reported as late as 4 months post-treatment initiation [24, 26–28].

When analysing potential predictors of endocrine toxicity in our cohort of patients with RCC under ICIs treatment, we found that patients who developed endocrine irAEs had significantly lower IMDC scores compared to those who did not. Notably, both PFS and OS at 12 months were significantly improved in patients with endocrine irAEs. These findings support existing evidence suggesting that the development of irAEs during ICI therapy is associated with more favourable clinical outcomes [29–36]. This association is likely due to the underlying mechanism of irAEs, which are thought to reflect an enhanced immune system activation, a mechanism also responsible for antitumor efficacy, thereby indicating a more robust therapeutic response to immune checkpoint blockade.

Interestingly, we observed a significantly lower incidence of endocrine irAEs in patients with renal/adrenal metastases. To the best of our knowledge, this is the first study to report such an association. Previous research has suggested that patients with adrenal metastases may exhibit poorer responses to ICI therapy compared to those with metastases in other sites [37–39]. The underlying mechanisms remain incompletely understood, but one plausible explanation involves the immunosuppressive tumour microenvironment characteristic of the adrenal glands. This microenvironment is modulated by endogenous factors such as glucocorticoids, catecholamines, and androgens, which may dampen immune activation and promote immune escape [40, 41]. Based on this rationale, it is conceivable that the reduced incidence of endocrine irAEs in these patients reflects a blunted immune response to ICIs, thereby aligning with a less favourable therapeutic outcome.

Unlike findings from larger studies involving diverse cancer populations [19, 24, 25, 27, 42–45], in our cohort, age, sex, and BMI were not identified as significant risk factors for the development of endocrine irAEs. This discrepancy may be attributed to the relatively small sample size of our study and the homogeneity of our patient population, which consisted exclusively of individuals with mRCC. In contrast, many of the aforementioned studies included patients with a variety of tumour types, potentially capturing broader risk patterns. Additionally, BMI data were not available for all patients in our cohort, which may have limited the power of our analysis to detect potential associations with this feature.

A subgroup analysis by sex revealed that, among male patients, endocrine irAEs were significantly more frequent in those with TNM stage II and III tumours and less common in those with TNM stage IV tumours. To the best of our knowledge, this is the first report to describe such a trend. A possible explanation may lie in the reduced effectiveness of ICI therapy in patients with more advanced or aggressive diseases, such as those with TNM stage IV tumours. In these cases, diminished immune activation may lead to both poorer clinical outcomes and a lower incidence of irAEs, reflecting the reduced engagement of the immune system. Currently, only a few studies have explored the relationship between tumour grade or cancer stage and the incidence of irAEs [46, 47], showing that in melanoma or lung cancer, higher disease burden is associated with increased risk of irAE, suggesting that these trends may be cancer-specific. Notably, the sex-related differences observed in the distribution of endocrine irAEs by TNM stage could be influenced by the immunomodulatory effects of sex hormones. Advanced tumors (i.e., stage IV) typically present with a highly immunosuppressive tumor microenvironment, limiting immune activation and reducing the likelihood of endocrine irAEs. In women, however, estrogens enhance immune responses, resulting in a more consistently activated immune system across disease stages, which may attenuate stage-related differences in irAE occurrence. It is important to note that the subgroup analysis by sex was exploratory, and the findings should be interpreted with caution given the wide confidence intervals observed for most estimates in the logistic regression analysis, reflecting limited sample size and sparse data in certain subgroups. Nonetheless, our results support the inclusion of sex–stage interaction terms in modeling. The distribution of irAEs across disease stages in men may represent a potential signal that warrants confirmation in larger, dedicated cohorts.

Previous studies have indicated that baseline blood cell counts (such as neutrophils, lymphocytes, monocytes, eosinophils, basophils, and platelets) as well as dynamic changes during treatment (e.g., increases in lymphocyte and eosinophil counts), and certain blood cell ratios (e.g., neutrophil-to-lymphocyte ratio), may be associated with a higher risk of developing irAEs [13]. In our cohort, we observed that endocrine irAEs were significantly more prevalent among patients with a pre-treatment neutrophil percentage below the 50th percentile. This finding aligns with prior reports suggesting that lower absolute neutrophil counts may serve as negative predictors of irAE development in patients receiving PD-1 inhibitors [48–50]. However, the strength of this association in our analysis was modest and should therefore be interpreted with caution.

As previously reported [19, 23, 28, 44, 51, 52], thyroid dysfunction, particularly hypothyroidism, was the most common endocrine irAE in our cohort, affecting 89.2% of patients who experienced endocrine toxicity, with 86.5% of these cases being hypothyroidism. We observed that patients treated with a combination of ICI and TKI developed thyroid dysfunction more frequently and with earlier onset compared to those receiving ICI + ICI or ICI combined with non-TKI anti-angiogenic agents. This finding is consistent with prior studies showing that thyroid dysfunction is a well-recognized adverse effect of TKI therapy [53–55] and is especially prevalent in patients treated with ICI + TKI combinations [56–58]. However, in contrast to previous findings, we did not observe a significant association between the presence of pre-existing antithyroid antibodies [14–16] or baseline TSH levels [17–20] and the occurrence of ICI-induced thyroid dysfunction. A likely explanation for this discrepancy is our exclusion of patients with pre-existing endocrine disorders, which may have led to an underrepresentation of individuals with elevated baseline TSH levels or positive antithyroid antibodies. Interestingly, like the broader category of endocrine irAEs, thyroid dysfunction was more frequently observed in patients with TNM stage II tumours and less commonly in those with stage IV tumours. This trend was confirmed in male patients but not in females, possibly due to the higher proportion of excluded female patients with pre-existing thyroid conditions, which resulted in a lower number of female patients included in the analysis.

Finally, in the logistic regression analysis using a stepwise model and adjusting for all previously identified significant parameters, the presence of renal/adrenal metastases and TNM stage IV tumours emerged as significant negative predictors of endocrine irAEs. Similarly, after adjustment for confounding variables, renal/adrenal metastases remained a significant negative predictor of thyroid adverse events, whereas TKI-based therapy was identified as a significant positive predictor.

The main limitations of our study are its relatively small sample size, retrospective monocentric design, and lack of external validation, which limit the generalizability of our findings and preclude definitive conclusions about causality. In addition, the retrospective nature of the study prevented us from distinguishing between different etiologies or the reversibility of irAEs, thus restricting the interpretability of our results. These limitations should be considered when interpreting the results. However, the study also presents several strengths. Notably, we included only patients without pre-existing endocrinopathies and with adequate follow-up, enhancing the reliability of endocrine irAE identification. Additionally, by focusing exclusively on patients with RCC, we minimized potential confounding effects related to tumour heterogeneity. Unlike many previous studies that primarily assessed irAEs in patients receiving ICI + ICI combinations, our cohort also included those treated with ICI + anti-angiogenic agents, allowing for a broader and more representative evaluation of current therapeutic strategies. Further prospective studies with larger patient cohorts are needed to validate our findings and to explore underlying mechanisms and predictive biomarkers of endocrine irAEs in this population.

In conclusion, our study reinforces the association between irAEs and improved clinical outcomes. Specifically, patients who developed endocrine irAEs demonstrated significantly better PFS and OS compared to those who did not, supporting the role of irAEs as potential biomarkers of favourable immunotherapy response. Notably, we identified for the first time the presence of renal/adrenal metastases and TNM stage IV tumours as significant negative predictors of endocrine irAEs. These findings suggest that tumour burden and aggressiveness may influence immune responsiveness and toxicity profiles. Further prospective, mechanistic studies are warranted to validate these associations and to elucidate the biological pathways underlying differential susceptibility to endocrine irAEs.

## Supplementary Information

Below is the link to the electronic supplementary material.

Supplementary Material 1

## Data Availability

Not applicable.
