# Supplementary material for: Risk factors for endocrinological immune-related adverse events in patients with renal cell carcinoma treated with immune checkpoint inhibitors
Source: J Endocrinol Invest. 2025 Nov 10;49(2):399–412. doi: 10.1007/s40618-025-02732-z (PMC12924852; doi:10.1007/s40618-025-02732-z)
Supplement: Supplementary file 1 — Supplementary Material 1 [file 40618_2025_2732_MOESM1_ESM.docx]

**Table S1. Multivariate logistic regression analysis evaluating the interaction between sex and stage disease for irAEs.**

| **Variables** | **Odds Ratio** | **95%CI** | **p-value** |
| --- | --- | --- | --- |
| **Male** | 0.174 | 0.02-0.99 | 0.056 |
| **Stage II** | 0.514 | 0.008-40.531 | 0.745 |
| **Stage III** | 0.086 | 0.002-4.303 | 0.196 |
| **Stage IV** | 0.286 | 0.009-8.310 | 0.41 |
| **Male * Stage II** | 5.833 | 0.149-159.78 | 0.297 |
| **Male * Stage III** | 25.667 | 1.872-449.202 | 0.018 |
| **Male * Stage IV** | NA | NA | NA |

CI: confidence interval; NA: not applicable

**Table S1. Multivariate logistic regression analysis evaluating the interaction between sex and stage disease for thyroid dysfunction.**

| **Variables** | **Odds Ratio** | **95%CI** | **p-value** |
| --- | --- | --- | --- |
| **Male** | 0.171 | 0.244-0.989 | 0.993 |
| **Stage II** | 8.04 | 3.349-NA | 0.993 |
| **Stage III** | 1.34 | 0.789-NA | 0.993 |
| **Stage IV** | 4.471 | 0.4-NA | 0.056 |
| **Male * Stage II** | 58.333 | 14.986-159.78 | 0.297 |
| **Male * Stage III** | 19.444 | 1.436-333.749 | 0.0301 |
| **Male * Stage IV** | NA | NA | NA |

CI: confidence interval; NA: not applicable
